# Pulmonary vascular disease associated with pulmonary hypertension in 445 patients: diagnosis from lung biopsy and autopsy

**DOI:** 10.1007/s11748-012-0155-7

**Published:** 2012-09-26

**Authors:** Shigeo Yamaki

**Affiliations:** Japanese Research Institute of Pulmonary Vasculature, 40-1 Usagisaku, Shiroishi, Miyagi 989-0228 Japan

**Keywords:** Pulmonary vascular disease, Pulmonary hypertension, Lung biopsy, Congenital heart disease, Chronic thromboembolic pulmonary hypertension

## Abstract

**Purpose:**

Diagnosis from lung biopsy or autopsy was performed in 445 patients with congenital (385) or acquired (60) heart disease from all over Japan. The purpose of this study is the presentation of these prospective data collections.

**Methods:**

Of the patients with congenital heart disease, 354 were biopsied to determine whether surgery was indicated. Decisions regarding surgery were based on the index of pulmonary vascular disease in simple cardiac anomalies or atrioventricular septal defects (AVSD). In total anomalous pulmonary venous connection (TAPVC), operative indication was determined by the degree of hypoplasia of small pulmonary arteries. Operability of Fontan procedure was based on the degree of residual medial hypertrophy after pulmonary artery banding.

**Results:**

In patients with simple cardiac anomalies, radical surgery was indicated in 166. Radical surgery was indicated in 50 patients with AVSD. In 26 patients with TAPVC, radical surgery was not indicated in 10. In 68 Fontan candidates, surgery was not indicated in 49. Among 7 patients with tetralogy of Fallot, 1 was not a surgical candidate. Of the 60 acquired heart disease patients, 16 had idiopathic pulmonary arterial hypertension and 36 had chronic thromboembolic pulmonary hypertension. In 6 patients, lung biopsy revealed pulmonary veno-occlusive disease; 2 patients had combined valvular disease.

**Conclusion:**

The cardiac surgeon, pediatric cardiologist, and cardiologist who requested diagnosis from lung biopsy or autopsy were gratified with the results.

## Introduction

I performed either lung biopsy or autopsy diagnosis in a total of 445 patients with pulmonary hypertension during the 5-year period from 2007 to 2011, as requested by the department of pediatric cardiology or cardiac surgery or cardiovascular medicine from all over Japan. The purpose of lung biopsy was to conclusively determine whether surgery or other treatment modalities were indicated. The purpose of autopsy was to conclusively determine in detail the exact cause of death in patients with pulmonary hypertension. Based on the pathological findings of pulmonary vascular disease, lung biopsies were assessed according to the diagnostic criteria for each disease; the diagnostic results are described in this study.

## Patients and methods

The 445 cases consisted of 385 cases of congenital heart disease and 60 cases of acquired heart disease. The breakdown is summarized in Tables 1 and 2.

**Table 1:** Congenital heart disease with pulmonary hypertension

	Mean age	Lung biopsy	Autopsy	Total
VSD and/or PDA and/or ASD	1 year 11 months	164	0	164
Atrioventricular septal defect	1 year 1 month	62	0	62
Simple ASD	9 years 11 months	27	0	27
TAPVC	2 months	26	13	39
Fontan candidate	7 years	68	0	68
Other CCA	1 year 1 month	0	10	10
Tetralogy of Fallot	7 years	7	0	7
IPPHN	3 months 10 days	4	4	8
Total		358	27	385

*VSD* ventricular septal defect, *PDA* patent ductus arteriosus, *ASD* atrial septal defect, *TAPVC* total anomalous pulmonary venous connection, *CCA* complicated cardiac anomalies, *IPPHN* idiopathic persistent pulmonary hypertension of the newborn

**Table 2:** Acquired heart disease with pulmonary hypertension

	Mean age	Lung biopsy	Autopsy	Total
IPAH	20 years	13	3	16
CTEPH	55 years	36	0	36
PVOD	11 years 2 months	5	1	6
Valvular heart disease	53 years	2	0	2
Total		56	4	60

*IPAH* idiopathic arterial pulmonary hypertension, *CTEPH* chronic thromboembolic pulmonary hypertension, *PVOD* pulmonary veno-occlusive disease

### Congenital heart disease

Of the 385 cases of congenital heart disease, 354 were lung biopsy cases to determine whether surgery was indicated, which excluded the following 3 diseases: 13 cases of total anomalous pulmonary venous connection (TAPVC), 10 cases of complex cardiac anomalies, and 8 cases of idiopathic persistent pulmonary hypertension of the newborn (IPPHN). Indication for lung biopsy in ventricular septal defect (VSD) and/or patent ductus arteriosus (PDA) and atrioventricular septal defect (AVSD) was determined based on the previously reported hemodynamic criteria. [1] In general, we evaluated whether surgery was indicated according to the index of pulmonary vascular disease (IPVD) [2] and the Heath–Edwards (HE) classification. [3] To obtain the IPVD score, the severity of each occlusive pulmonary vascular lesion in the small pulmonary arteries was categorized as grade 1–4 and an arithmetic average for all lesions was calculated in each case. The indication for surgery differed slightly by primary disease. Whether surgery was indicated for simple atrial septal defects (ASD) was evaluated according to the previously reported criteria [4], because systemic arterial pressure is not directly applied to the pulmonary arteries, unlike in other simple cardiac anomalies such as VSD or PDA. For simple cardiac anomalies not associated with simple ASD, surgery was indicated for IPVD ≤ 2.0 in patients without Down syndrome and ≤2.1 in patients with Down syndrome [5]. Surgery was indicated for patients with AVSD with IPVD ≤ 2.0 [6]. Patients with absolute operative contraindication [7] were not considered candidates for surgery. When an extremely thickened media was observed in ≥10 % of all small pulmonary arteries, surgery was not indicated [8]. For simple ASD, whether surgery was indicated depended on the presence or absence of 4 pathological conditions [4]. In particular, patients with ASD associated with the following were considered candidates for surgery: IPVD ≤ 2.2 for the plexogenic arteriopathy type, musculoelastosis [9], pulmonary thrombosis, or the mixed type where no collateral circulation (e.g. plexiform lesions) was observed. When wall thickness was greater than 40 %, we considered the case to be associated with idiopathic pulmonary arterial hypertension (IPAH).

In cases of lung biopsy, we categorized the lung biopsy results into 1 of the following 5 categories by comprehensively taking into account the pathological findings along with the status of the respiratory system and pulmonary veins.

A. Absolutely no postoperative problems.
B. No operative or in-hospital death, but pulmonary hypertension remains.
C. No operative or in-hospital death, but late death is possible.
D. No operative or in-hospital death, but late death is likely.
E. Operative or in-hospital death.

In cases of TAPVC, where hypoplasia of the small pulmonary arteries significantly affects the prognosis [10], categorization was performed based on the diameter of the small pulmonary arteries parallel to the adjacent terminal bronchi: A, diameter ≥80 % of normal; B, 60–80 %; C, 40–60 %; D, 20–40 %; and E, ≤ 20 %. Of note, we rarely found obstructive pulmonary vascular lesions.

The Fontan procedure was considered to be indicated if the following were present: (1) complete normalization of the hypertrophy of the media in all of the intraacinar small pulmonary arteries and (2) complete normalization of the hypertrophy of the media in ≥50 % of the preacinar small pulmonary arteries after pulmonary artery banding (PAB) [11]. Each case was categorized as A, B, or C depending on the degree of pulmonary arterial normalization. Moreover, patients with normalization of the hypertrophy of the media in all the intraacinar small pulmonary arteries but ≤50 % of the preacinar small pulmonary arteries were categorized as D. Finally, cases where hypertrophy of the media in both the preacinar and intraacinar small pulmonary arteries remained were categorized as E.

### Acquired heart disease

For acquired heart disease, we diagnosed a total of 16 cases of idiopathic pulmonary arterial hypertension (IPAH) including 9 cases where lung tissue was collected during lung transplantation, 4 cases associated with ASD in which operability was asked, and 3 autopsy cases. IPAH cases were categorized as either the juvenile type, where hypertrophy of the media advanced in the early stage, or the adult type where the pulmonary vascular obstructive episode advanced in early stage [12]. The juvenile type was mainly treated with pulmonary vasodilators. Lung biopsy for chronic thromboembolic pulmonary hypertension (CTEPH) was performed to evaluate the perioperative and long-term postoperative prognosis. Valvular heart disease patients were all considered to be candidates for surgery in the absence of pulmonary veno-occlusive disease (PVOD).

Preparation of pathological specimens was performed as follows: 30 pieces of step sections (with a 50 μm step) were prepared to facilitate the pathological diagnosis. Goldner’s trichrome stain combined with Weiger’s stain for elastic fibers was performed [1, 2, 4–14].

## Results

### Congenital heart disease

The breakdown of the 385 congenital heart disease cases was as follows: 164 cases of simple cardiac anomalies excluding simple ASD (including 94 cases of Down syndrome); 62 AVSD (including 56 Down syndrome); 27 ASD (including 6 Down syndrome); 39 TAPVC (including 13 autopsy cases); 68 cases of complex cardiac anomalies (Fontan candidates); 7 tetralogy of Fallot (TOF) associated with pulmonary hypertension and 8 IPPHN (including 4 autopsy cases); and 10 autopsy cases of complex cardiac anomalies.

#### Simple cardiac anomalies (VSD and/or PDA and/or ASD)

Lung biopsy was performed in all 164 cases of simple cardiac anomalies. Patients ranged in age from 1 month to 58 years (mean, 23 months). Lung biopsy specimens were collected preoperatively in 54 patients, during pulmonary artery banding (PAB) in 96 patients, intraoperatively in 13 patients, and during PDA ligation in 1 patient.

Pathological examination revealed that radical surgery was indicated in 145 patients (A, 62; B, 65; C, 18), but not indicated in 19 patients (D, 9; E, 10). Patients evaluated as D included 3 with an extremely thickened media in the small pulmonary arteries (4, 5, and 7 months old). When the 4-month-old patient underwent lung biopsy again at the age of 18 months after PAB, the extremely thickened media of the small pulmonary arteries reduced, and the patient’s status improved to A. Hypoplasia of the small pulmonary arteries was identified in 3 patients with Down syndrome (2, 4, and 4 months old) and they were evaluated as D. In a 34-month-old patient, smooth muscle cells were completely absent, leading to a diagnosis of hypoplasia of the small pulmonary arteries and evaluation as D. Hypoplasia of the respiratory system was also observed in 1 Down syndrome patient (4 months old) evaluated as D. In addition, an impending absolute operative contraindication was found in a 29-year-old evaluated as D. There were 9 patients evaluated as E, including 4 patients with Down syndrome (1, 3, 6, and 11 months old) and 5 without Down syndrome (1, 3, 4, 6, and 7 months old). Of these 5 patients with absolute operative contraindication, in the 4-month-old, a part of complete obstructive pulmonary vascular lesions disappeared 1 year after PAB; likewise in the 7-month-old patient, similar changes were observed 2 years after PAB. Both ultimately underwent radical surgery. In addition, 1 patient with extremely thickened media of small pulmonary arteries was evaluated as E.

Of the 164 cases in which lung biopsy was performed, 94 were associated with Down syndrome (A, 38; B, 41; C, 6; D, 4; E, 5). No significant differences were found between Down syndrome and non-Down syndrome patients. The severity of the obstructive pulmonary vascular lesions was assessed according to the HE classification (I, 78; II, 48; III, 36; IV, 2). IPVD ranged from 1.0 to 3.0 (mean, 1.2). There were 3 patients with IPVD of 2.2, 2.9, and 3.0, exceeding the criteria for surgery.

#### AVSD

There were 62 patients with AVSD, whose ages ranged from 14 days to 10 years (mean, 13 months). Of these 62 patients, 56 were associated with Down syndrome. Lung biopsy specimens were collected during PAB intraoperatively, and preoperatively in 44, 4, and 3 patients, respectively. The remaining 11 patients had previously undergone PAB.

Radical surgery was indicated in 50 patients (A, 18; B, 27; C, 5) but not in 12 patients (D, 8; E, 4). The latter 12 cases were all associated with Down syndrome. The 8 cases evaluated as D were as follows: 4 patients had an extremely thickened media in the small pulmonary arteries (1, 6, 7, and 12 months old), 2 patients had impending absolute contraindications to surgery (3 and 6 months old), and 2 patients had IPVD ≥ 2.1 (IPVD 2.1 in a 6-month-old and 2.5 in an 8-year-old, respectively). Among the 4 patients evaluated as E, 3 had absolute operative contraindication (3, 4, and 42 months old) and 1 had extremely thickened media of small pulmonary arteries (1 month old).

The HE grade was I in 38 patients, II in 15, III in 9, and IV in 0. IPVD ranged from 1.0 to 2.5 (mean, 1.2).

#### ASD

There were 27 patients with ASD whose ages ranged from 2 months to 45 years (mean, 9 years 11 months); 6 patients had Down syndrome.

The breakdown of ASD was as follows: 16 patients with plexogenic arteriopathy, 7 with IPAH, 3 with musculoelastosis, and 1 with an extremely thickened media in the small pulmonary arteries. There were no cases associated with pulmonary thrombosis.

Radical surgery was indicated in 21 patients (A, 2; B, 8; C, 11), but not in 6 patients (D, 3; E, 3). Of the 3 patients evaluated as D, 2 had IPAH (9 months, 22 years old) and 1 had advanced plexogenic arteriopathy with an IPVD of 2.8. The 3 patients evaluated as E included 1 patient with ASD associated with juvenile IPAH (21 years old), 1 with an extremely thickened media in the small pulmonary arteries (8 months old), and 1 with Eisenmenger syndrome (22 years old). The patient with Eisenmenger syndrome had advanced plexogenic arteriopathy (IPVD, 3.1) and many plexiform lesions were observed as collateral circulation.

The HE grade was I in 10 patients, II in 5, III in 8, and IV in 4. IPVD ranged from 1.0 to 3.1 (mean, 1.3).

#### TAPVC

Of these 39 patients with TAPVC, initial radical surgery was performed in 20 patients (0 days to 3 months old; mean, 10.5 days). To remove the pulmonary venous obstruction (PVO), a second surgery was performed in 6 patients (3–6 months old; mean, 3.7 months). Autopsy was performed in 13 cases (17 days to 22 months old; mean, 5.2 months). Lymphangiectasia was observed in 16 cases (41 %).

The 20 patients who had radical surgery were rated as follows A, 0; B, 5; C, 8; D, 6; and E; 1. The 6 patients who had a second surgery were evaluated as follows: B, 1; C, 2; and D, 3. The 13 autopsy cases included 6 cases of D and 7 cases of E. Of these 13 autopsy cases, the cause of death was PVO in 12 cases and an extremely thickened media in the small pulmonary arteries in 1 case. Severe hypoplasia of the small pulmonary arteries was observed in 6 cases. There was 1 case where the media in the small pulmonary arteries was completely absent. The HE grade in patients with initial radical surgery was I in 16 patients, II in 1, III in 1, and IV in 2 (angiitis) and IPVD was 1.0 in 16 patients, 1.1 in 1, 1.2 in 2, and 1.6 in 1. In the 6 cases where lung biopsy was performed during the second surgery to remove the PVO, the HE grade was I in 3 patients, II in 2, and III in 1, and IPVD was 1.0 in 3 patients, 1.1 in 2, and 1.6 in 1. In the 13 autopsy cases, the HE grade was I in 12 cases and III in 1 (longitudinal smooth muscle bundle). IPVD was 1.0 in 11 cases and 1.1 in 1. IPVD was not evaluable in the case with longitudinal smooth muscle bundles.

#### Complex cardiac malformations in Fontan candidates

The primary disease in Fontan candidates was as follows: 21 cases of single ventricle (SV) (0 days to 26 years; mean, 4.4 years), 14 of double outlet right ventricle (DORV) (1 day to 16 years; mean, 4.2 years), 9 of hypoplastic left heart syndrome (HLHS) (0 days to 8.7 years; mean, 1.6 months), 7 of AVSD (1 month to 7 years; mean, 2 years), 5 of tricuspid atresia (TA) (1 year to 28 years; mean 5.2 years), 4 of transposition of the great arteries (TGA) (1 month to 16 years; mean, 6.6 years), and 8 other cases. In these 68 Fontan candidates, PAB was performed in 57 patients, but not in 11 patients with preexisting pulmonary stenosis (PS). The HE grade was 0 (no hypertrophy of the media of the small pulmonary arteries) in 10 patients, I (only hypertrophy of the media was identified) in 34, II in 5, III in 10, and IV in 2. The HE grade could not be determined in 7 cases due to thrombi. IPVD ranged from 1.0 to 2.8 (mean, 1.2) in 61 cases, excluding 7 cases where thrombi were present.

Lung biopsy specimens were collected in 11 patients during PAB, 47 during the Glenn procedure, 6 during re-PAB, 3 of open lung biopsy performed after the Glenn procedure, and 1 during the Fontan procedure. In the 11 patients whose lung biopsy specimens were collected during PAB, age ranged from 0 days to 6 months (mean, 1.6 months). Since these 11 patients had hypertrophy of the media, there was no A, B, or C ratings. Lung biopsy resulted in 5 patients evaluated as D and 6 as E. In the 47 patients in whom lung biopsy was performed during the Glenn procedure, age ranged from 19 days to 18 years (mean, 3.7 years). Lung biopsy resulted in 5 patients rated as A, 5 as B, 7 as C, 26 as D, and 4 as E. In the 6 patients in whom lung biopsy was performed during re-PAB, 4 were evaluated as D and 2 as E. The 3 patients who underwent open lung biopsy after the Glenn procedure included 2 patients rated as C and 1 as D. The patient whose lung biopsy specimen was obtained during the Fontan procedure was evaluated as D, and the Fontan was subsequently taken down due to insufficient postoperative pulmonary blood flow.

#### Autopsy in cases of complex cardiac malformations

There were 10 autopsy cases associated with congenital heart disease, including 4 HLHS (1, 4, 6, and 12 months old), 4 SV (1, 5, and 21 months, and 15 years old), 1 DORV (11 months old), and 1 TGA (1 month old).

#### TOF

There were 7 patients with TOF associated with pulmonary hypertension whose ages ranged from 7 months to 37 years (mean, 7 years). Shunt surgery was thought to be the cause of pulmonary hypertension in all of these patients. They were evaluated as follows: A in 2 patients, B in 3, C in 1, and D in 1. In the 5 patients categorized as A or B, mild intimal fibrous hypertrophy was observed and radical surgery was indicated. On the other hand, the 2 patients categorized as C or D with proliferation of the longitudinal muscle cells had previously undergone central shunt surgery. Radical surgery was not indicated in 1 of 2 because of severe occlusion of the longitudinal smooth muscle cells in small pulmonary arteries.

#### IPPHN

There were 8 patients with IPPHN (5 days to 6 months old). Of these, 4 were autopsy cases. No obstructive pulmonary vascular lesions were identified and all were categorized as HE I and IPVD 1.0. The patients had been treated with nitric oxide or bosentan since birth, and histopathological examination revealed extensive hypoplasia in the respiratory system and also in the small pulmonary arteries.

### Acquired heart disease

The 60 patients with acquired heart disease included 16 with IPAH (3 autopsy cases), 36 with CTEPH, 6 with PVOD (1 autopsy case), and 2 with acquired combined valvular disease.

#### IPAH

Of the 16 patients clinically diagnosed with IPAH, 7 had a juvenile type and 9 had an adult type, and ages ranged from 15 months to 54 years (mean, 20 years). The 3 autopsy cases included a 27-year-old patient who died from scirrhous complications, a 10-year-old patient who died from respiratory failure including atelectasis, and a 19-year-old patient who died from complications of advanced obstructive pulmonary vascular disease including angiitis. In 4 patients with IPAH associated with ASD, lung biopsy was performed to determine whether surgery was indicated. These patients had a juvenile type (11 and 11 months, and 16 and 25 years), with progressive medial hypertrophy more than 40 % in wall thickness. In 2 patients with IPVD 1.0, ASD closure deemed possible with the use of vasodilators and 1 patient underwent surgery. The other 2 patients had high IPVD (2.3 and 2.8) and thus ASD closure was not performed in order to maintain a safety valve for the right-to-left shunt.

Lung transplantation was performed on 9 patients, ranging in age between 11 and 54 years. There were 8 cases of the adult type, where IPVD was between 2.7 and 3.2 (mean, 3.0) and advanced obstructive pulmonary vascular lesions, including plexiform lesions, were observed.

#### CTEPH

In the 36 patients with CTEPH, intraoperative lung biopsy was performed to evaluate both the effects of the endarterectomy and prospects for long-term postoperative quality of life as well as to provide patients with guidance regarding daily life activities. Their ages ranged from 28 to 75 years (mean, 55 years). There were 30 patients with New York Heart Association grade III and 6 with IV. The mean pulmonary arterial pressure of each patient ranged from 28 to 65 mmHg (overall mean, 46 mmHg). After lung biopsy, 12 patients were evaluated as A, 10 as B, 6 as C, and 8 as D. In all the patients rated D, the pulmonary artery was occluded by many layers of aged thrombi. The old emboli were not recanalized and the media in the peripheral small pulmonary arteries were atrophied. Of particular note in all of 36 patients, proliferation of collagen fibers was observed in all of the pulmonary veins, similar to findings in PVOD. Some of the pulmonary veins were more than 75 % occluded, suggesting that hemodynamics would be affected.

#### PVOD

There were 5 patients with PVOD who underwent lung biopsy (3 months to 6 years old). All of them had severe pulmonary hypertension. The pulmonary veins were occluded by collagen fibers. Severe mitral regurgitation was found in an autopsy case of a 48-year-old patient.

#### Combined valvular heart disease

There were 2 patients with combined valvular heart disease who underwent lung biopsy (53 and 54 years old). Both were evaluated as B.

## Discussion

Deciding whether radical surgery is indicated in patients with congenital heart disease is not straightforward and depends on the type of disease. Based on our past experience, we have been evaluating whether surgery is indicated in patients with various congenital heart diseases based on IPVD scores [2]. In patients with simple cardiac anomalies such as VSD where the pulmonary arteries are directly subjected to systemic arterial pressure, surgery is indicated when IPVD ≤ 2.0 in patients without Down syndrome and IPVD ≤ 2.1 in patients with Down syndrome [5]. These criteria were used in the present report; however, no differences were found in the severity of obstructive pulmonary vascular lesions between these two patient populations, probably because the mean age was low (23 months). Absolute operative contraindications were observed as early as the age of 1 month in patients without Down syndrome and 3 months in patients with Down syndrome. This is why lung biopsy is important even in patients as young as 1 month. In all patients, it was expected that PAB would cause blocking and involution of obstructive pulmonary vascular lesions. In particular, radical surgery was indicated 1 year after PAB in a 4-month-old and 2 years after PAB in a 7-month-old; both did not have Down syndrome, but had the finding of absolute operative contraindication. They underwent surgery safely.

Of the 3 patients with extremely thickened media of small pulmonary arteries where radical surgery was not indicated, there was 1 patient where surgery was indicated when he was re-biopsied 14 months after PAB; this patient underwent radical surgery. This demonstrates that patients with extremely thickened media of small pulmonary arteries can become candidates for surgery after PAB.

All 12 patients with AVSD who were not deemed candidates for radical surgery had Down syndrome demonstrating that it aggravates obstructive pulmonary vascular lesions. Radical surgery was not indicated in 19 % of all AVSD patients in the present report, which is approximately twice the 10 % rate of inoperable patients with simple cardiac anomalies.

In most patients with AVSD as well as simple cardiac anomalies, lung biopsy was performed during PAB. A two-stage procedure helped prevent mortality due to obstructive pulmonary vascular lesions, indicating that this procedure was highly recommended [13].

There were 8 ASD patients with associated IPAH; surgery was indicated in the 5 patients where ASD closure was performed and pulmonary hypertension was improved by medication postoperatively. In 1 patient with plexogenic arteriopathy evaluated as E, IPVD was 3.1, and collateral circulation, such as in plexiform lesions, was complete, leading to a diagnosis of Eisenmenger syndrome. Since the prognosis of Eisenmenger syndrome associated with ASD is good even without treatment, this patient was followed with observation.

Since the mean age of the 20 patients with TAPVC who underwent radical surgery was 10.5 days, obstructive pulmonary vascular lesions were not present. In cases where a second surgery or autopsy was performed, IPVD was ≤1.6 and surgery was indicated in all cases since obstructive pulmonary vascular lesions were under the operative indication. However, in 7 patients who underwent an initial surgery, there was significant hypoplasia of the small pulmonary arteries. As we previously reported, hypoplasia of small pulmonary arteries can result in death if patients do not have sufficient pulmonary blood flow after surgery [14]. Therefore, when an ASD is small (≤3 mm) and hypoplasia of the small pulmonary arteries is suspected in patients with TAPVC, it is necessary to retain or enlarge the ASD. If hypoplasia of the small pulmonary arteries improves and sufficient pulmonary blood flow is maintained after surgery, then the ASD can be closed.

In 13 autopsy cases of TAPVC, the most common cause of death was PVO (12 cases), indicating that postoperative PVO is a serious issue. It had previously been reported that lymphangiectasia was observed in 30 % of patients with TAPVC [10], but in the present study a higher (40 %) frequency of lymphangiectasia was observed.

The most common primary disease in Fontan candidates was SV, but there were also many patients with DORV, AVSD and TA where the Fontan procedure was determined to be the best treatment given the hypoplasia of the left and right ventricles. In patients without PS, PAB must be performed. In the present report, lung biopsy was performed even during PAB in such patients, but biopsy was not very useful because hypertrophy of the media was advanced. Instead, lung biopsy performed during the Glenn procedure was mainly used to determine whether the Fontan procedure was indicated. Of the 47 patients where lung biopsy was performed during the Glenn procedure, 30 were considered not to be candidates for the Fontan procedure. These 30 patients had high pulmonary arterial pressure and pulmonary vascular resistance, and lung biopsy was performed as the final decision of Fontan operation. These patients are currently awaiting normalization of hypertrophy of the media with medication.

In patients with TOF, a Blalock–Taussig (BT) shunt is usually created. If sufficient pulmonary blood flow is not obtained after this procedure, a central shunt may be created to directly connect the right ventricle to the pulmonary trunk using a more than 5-mm artificial graft. However, the present study revealed that advanced hypertrophy of the longitudinal smooth muscle cells can occlude the pulmonary arteries and thus a central shunt should be avoided [15].

In IPPHN, hypoplasia of the respiratory system has been pointed out and patients have been treated with high frequency jet ventilation and extracorporeal membrane oxygenation. However, the prognosis remains extremely poor. According to the findings in the present report, extensive hypoplasia was observed also in the small pulmonary arteries, suggesting that IPPHN can be treated with a BT shunt to improve pulmonary blood flow.

Plexogenic arteriopathy was observed in all cases of IPAH. In all patients who were candidates for lung transplantation, collateral circulation, such as plexiform lesions, was complete and thus it was thought that lung transplantation was the only treatment option.

In contrast, in the 2 juvenile type IPAH patients with ASD, there were no advanced obstructive pulmonary vascular lesions and hypertrophy of the media. It was thought that in these patients, ASD closure could be achieved with vasodilators. In patients with ASD, even those with advanced obstructive pulmonary vascular lesions, ASD could work as a safety valve for the right-to-left shunt, suggesting that such patients could achieve long-term survival.

In CTEPH, it is presumed that the presence of thrombi and the severity of lesions may vary from site to site in the lung. However, evaluating the degree of occlusion in the small pulmonary arteries via lung biopsy was effective in determining whether sufficient blood flow to the peripheral arteries was maintained after endarterectomy.

Although PVOD is rarely seen in children, 5 cases of PVOD were found in children younger than 6 years old. Although all of them had advanced pulmonary hypertension, clinical examinations such as cardiac catheterization could not determine the diagnosis of PVOD. Therefore, lung biopsy was performed. In particular, neoplasia of the collagen fibers in the small pulmonary veins occluding the lumen was observed, causing advanced pulmonary venous hypertension.

## Conclusion

We examined lung biopsy and autopsy specimens in 445 patients with pulmonary vascular disease associated with pulmonary hypertension, including 385 patients with congenital disease and 60 with acquired disease. Of the 385 congenital heart disease patients, lung biopsy was performed in 354 to determine whether surgery was indicated. Of the 60 acquired heart disease patients, there were 16 with IPAH. Lung biopsy was performed in 36 patients with CTEPH during endarterectomy, which was effective in evaluating the long-term postoperative quality of life. In addition, there were 6 patients with PVOD and 2 with combined valvular disease.

Although in this study, prospective data were collected in patients with pulmonary hypertension, the cardiac surgeon, pediatric cardiologist and cardiologist who requested lung biopsy and autopsy for diagnosis of pulmonary vascular disease were gratified with the results. However, a retrospective study which includes long-term postoperative prognoses is needed in the near future.
